# The roles of nasal nitric oxide in diagnosis and endotypes of chronic rhinosinusitis with nasal polyps

**DOI:** 10.1186/s40463-020-00465-y

**Published:** 2020-09-22

**Authors:** Mengdi Zhu, Xuehuan Gao, Zhuang Zhu, Xiaoqing Hu, Hui Zhou, Jisheng Liu

**Affiliations:** 1 Department of Otorhinolaryngology Head and Neck Surgery, The First Affiliated Hospital of Soochow University, Shi’zi Road 188, Suzhou, 215006 China; 2 Department of Environmental Health, School of Public Health, Fudan University, Shanghai, China; 3 Department of Orthopaedics, The First Affiliated Hospital of Soochow University, Suzhou, China

**Keywords:** Chronic rhinosinusitis, Nasal polyps, Nasal nitric oxide, Eosinophil

## Abstract

**Background:**

Chronic rhinosinusitis with polyps (CRSwNP) is a global health concern. Nasal nitric oxide (nNO), a clinical biomarker, have been studied to assess the presence of airway mucosal inflammation. This study aimed to clarify the roles of nNO in diagnosis and endotypes of CRSwNP.

**Methods:**

Eighty-two CRSwNP patients and thirty healthy volunteers were recruited for this study. The patients were classified into eosinophilic CRSwNP (Eos CRSwNP) and non⁃eosinophilic CRSwNP (Non-Eos CRSwNP) endotypes by tissue eosinophil percentage. nNO levels were measured with an electrochemical sensor-based device. nNO levels and clinical factors were compared among the groups. Receiver-operating characteristic (ROC) curve and logistic regression analyses were performed to evaluate the predictive ability of the nNO for diagnosis and endotypes of CRSwNP.

**Results:**

Eos CRSwNP patients(143.9 ± 106.2, ppb) had lower nNO levels than Non-Eos CRSwNP(228.3 ± 109.2, ppb, *p* = 0.013) and healthy subjects(366.5 ± 88, ppb, *p* < 0.0001). Patients with atopy exhibited significantly higher levers of nNO compared with patients without atopy (*p* < 0.05). For Eos CRSwNP diagnosis, nNO had high predictive value for Eos CRSwNP (AUC: 0.939; sensitivity: 76.74%; specificity: 96.67%; cut-off value: 231 ppb, *p* < 0.001). Furthermore, nNO levels were associated with CRSwNP endotypes (odds ratio: 1.010; 95% confidence interval: 1.003, 1.016%; *p =* 0.002). When the nNO concentration was 158 ppb, we could discriminate Eos CRSwNP from Non-Eos CRSwNP (AUC = 0.710, sensitivity: 76.92%; specificity, 60.47%, *P* = 0.001). After it was combinated by nNO, peripheral blood eosinophil count (PEAC) and VAS score, the AUC was increased to 0.894 (95%CI = 0.807 to 0.951, *p* < 0.0001, sensitivity:76.74%, specificity: 89.74%).

**Conclusions:**

nNO may have potential for non-invasive diagnosis and endotype of CRSwNP. nNO combined with PEAC and VAS score may be a good diagnostic tool for endotyps of Eos CRSwNP. However, the atopic status of the patients influenced the levels of nNO.

## Introduction

Nitric oxide (NO) is an endogenous mediator produced by L-arginine and oxygen through activity of nitric oxide synthase (NOS). NOS is present in constitutive or inducible forms, with inducible NOS (iNOS) considered of fundamental importance for inflammatory processes. Increased iNOS leads to an increase of NO levels in response to inflammatory stimuli. Measurements of NO can be divided into two main categories: fractional exhaled NO (FeNO) and nasal NO (nNO) [1]. FeNO, a non-invasive immune biomarker, has become a routine test item in diagnosis and treatment of asthma [2]. Likewise, nNO can potentially provide a rapid, low cost objective measure of lower airway inflammation [3], but it is not widely used in the field of rhinology. Furthermore, the levels of nNO in different endotypes of chronic rhinosinusitis with nasal polyps (CRSwNP) remain unclear.

CRSwNP is characterized by the accumulation of inflammatory cells. However, an eosinophil predominance is seen in western countries, but not Asian, patients with polyps [4]. Therefore, some scholars have proposed that CRSwNP can be divided into two endotypes based on the degree of eosinophil infiltration into the nasal mucosa: eosinophilic CRSwNP (Eos CRSwNP) and non-eosinophilic CRSwNP (Non-Eos CRSwNP) [5]. Compared with Non-Eos CRSwNP, Eos CRSwNP was shown to be frequently associated with extensive olfactory dysfunction [6], higher incidence of asthma [7], greater sensitivity to glucocorticoid therapy [8], higher recurrence rates, and higher computed tomography (CT) score [9]. The gold standard for the classification of eosinophilic and non-eosinophilic CRSwNP is histologic criteria. Whereas, use of this markers is limited because it is impossible or invasive to obtain before surgery. Accordingly, it is highly desirable to seek a simple predictor. In recent years, several clinical parameters like peripheral blood eosinophil count (PEAC) and CT score have been used for predicting the endotypes of CRSwNP [10, 11]. Similarly, nNO may be a simple, rapid, well-tolerated, and noninvasive predictor in the clinical setting.

Recently, Yoshida et al. [12] reported that decreased exhaled nitric oxide (NO) may be a good predictor of eosinophilic chronic rhinosinusitis in the Japanese population. However Takeno et al. [13] described the opposite findings. Moreover, the definition of Eos CRSwNP in these studies was based on clinical manifestations. Whereas, based on the range of normal values, Cao et al. [14] have identified that Eos CRSwNP was defined when the percentage of eosinophils in tissues exceeded twice the standard deviation of the mean percentage in control tissues, with 10% calculated as the cut-off value. The rationality of this method have been proved by several studies [15–17]. Based on this method, the aim of the present study was therefore to investigate the predictive value of the nNO for diagnose and differentiate between Eos CRSwNP and Non-Eos CRSwNP.

## Patients and methods

### Subjects

The study was approved by the Ethics Committee of the First Affiliated Hospital of Soochow University and informed consents were obtained from all subjects before enrollment. Eighty-two CRSwNP patients and thirty normal controls were recruited into this cross-sectional study from November 2018 to May 2019. The diagnosis of CRSwNP was made according to the European Position Paper on Rhinosinusitis and Nasal Polyps 2012 [18]. The exclusion criteria were pregnancy, children under 18 years of age, systemic severe diseases, acute upper or lower respiratory tract infections within 2 weeks before the visit, and use of specific medications (oral or local corticosteroids, antihistamines, leukotriene receptor antagonist and antibiotics) within 4 weeks before the inclusion visit. Allergic rhinitis (AR) was diagnosed by positive skin prick test (SPT) responses and/or prior physician diagnosis and clinical history. Positive SPT was defined as an allergen-histamine-induced wheal of 3 mm. All SPTs were administered and interpreted by a rhinologist, who was blinded to the patient’s clinical status, and atopy was defined as the presence of positive SPT reaction to at least one of the aeroallergens. Asthma was diagnosed by pulmonary function studies or prior diagnosis by a pulmonologist. Major nasal symptoms including nasal obstruction, nasal discharge, olfactory dysfunction, facial pain, and headache were scored on a visual analogue scale (VAS) of 0 to 10, with 0 being no complaints at all and 10 being the worst imaginable. Patients were also asked to rate their overall burden of CRS symptoms. All patients underwent a CT scan of the paranasal sinuses before surgery. The CT scan was scored by the Lund–Mackay scoring system as follows: 0, no opacification; 1, partial opacification; 2, complete opacification; ostiomeatal complex score of 0, not occluded and 2, occluded. The score on each side was scaled up to range from 0 to 12, giving a total range of 0 to 24, with higher scores indicating a worse status. The ratio of the ethmoid sinus and maxillary sinus CT score (E/M ratio) was also recorded. Preoperative endoscopic scoring was performed by the Lund–Kennedy scoring system. For this, the polyp score were: 0, no polyps; 1, polyps in the middle meatus, but not reaching below the inferior border of the middle turbinate; 2, polyps beyond the middle meatus. A complete peripheral blood cell count with differential was performed by automated analysis, and the eosinophil percentage and absolute eosinophil count were calculated.

### Subclassification of CRSwNP

**Fig. 1:**
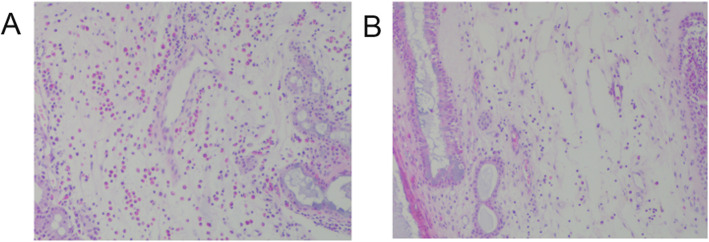
Histological assessment of mucosa in Eos CRSwNP (**a**) and Non-Eos CRSwNP (**b**) (H&E, × 200 magnification). **a** A representative sample from tissue of a patient with Eos CRSwNP patient demonstrating infiltration by a large number of eosinophils and a severely edematous basement membrane. **b** A representative sample from tissue of a patient with Non-Eos CRSwNP patient demonstrating infiltration by large numbers of neutrophils. Eos CRSwNP = eosinophilic chronic rhinosinusitis with nasal polyps. Non-Eos CRSwNP = non eosinophilic chronic rhinosinusitis with nasal polyps

All patients underwent functional endoscopic sinus surgery (FESS), and nasal polyps tissues obtained during surgery. All samples were stained with hematoxylin and eosin stain and then observed at 400× magnification for the numbers of infiltrating inflammatory cells, including eosinophils, neutrophils, and lymphocytes. The total number and mean number of eosinophils in five fields were assessed by two independent pathologists who were blinded to the patient’s information. The percentage of eosinophils in the total inflammatory cells was recorded. Eos CRSwNP was defined as percentage of tissue eosinophils > 10% in the total infiltrating cells [19], while Non-Eos CRSwNP was defined as percentage ≤ 10% (Fig. 1).

### Measurements of nNO

Levels of nNO were measured with a NIOX MINO (Aerocrine AB, Solna, Sweden), an online NO testing instrument, based on the American Thoracic Society/European Respiratory Society guidelines [2]. The measurement unit was parts per billion (ppb). The 44-s test was administered at a time between 1:00 and 4:00 pm, with room temperature maintained at 16–30 °C and relative humidity maintained at 20–60%. Patients were required to avoid smoking, eating, drinking, and strenuous exercise for 1 h before the measurement. The affected nostrils were blocked by a nasal olive with a central lumen connected to the analyser, making a tight seal while the contralateral nostril was left open. Transnasal airflow was applied at a constant flow rate of 5 ml/s. To isolate the nasal cavity from lower airway, velopharyngeal closure was achieved through the inhalation to total lung capacity, and oral exhalation maintaining an expiratory pressure of > 10 cm H_2_O separates the nasal cavity from the lower airway. The mean nNO level of the two nostrils was determined after performing three exhalations at 15 min aintervals.

### Statistical analysis

All data plots and statistical analyses were conducted using IBM SPSS Statistics for Windows Version 21.0 (IBM Corporation, Armonk, NY) and Prism 8 (GraphPad, La Jolla, CA) software. The patients were divided into entire group and subgroups based on patient factors. For continuous variable, comparisons within multigroup were performed with the Kruskale-Wallis 1-way ANOVA with Bonferroni post-hoc test, and comparisons between 2 groups were performed with the Student’s *t* test or Mann-Whitney *U* test according to 2 main assumptions (normality and homogeneity of variance). Categorical variables were analyzed with χ2 test or Fisher’s exact test. Measurements were expressed as medians and ranges. Logistic regression models were applied to assess the relationships between predictor variables and endotypes of CRSwNP. The odds ratio (OR) and 95% confidence intervals (95%CI) were calculated for each parameter. Correlations were assessed by using Spearman rank correlation. A receiver-operating characteristic (ROC) curve analysis was performed to evaluate the potential of nNO for diagnosis or endotypes of CRSwNP, with an area under the curve (AUC) value close to 1 indicating high predictability. The optimal cut-off value was determined by the Youden index (sensitivity + specificity – 1) based on the ROC curve. Values of *p* < 0.05 were considered to indicate statistical significance.

## Results

### Demographic and clinical characteristics of the subjects

The demographic characteristics of the patients are shown in Table 1. Based on the histological criteria for the 82 CRSwNP patients, 43 were classified into the Eos CRSwNP group and 39 were classified into the non-Eos CRSwNP group. The subjects were further divided into being atopic and nonatopic groups. The following clinical manifestations were significantly higher in the Eos CRSwNP than in the Non-Eos CRSwNP group: tissue eosinophils count (*p* = 0.001) and tissue eosinophils percentage (*p* = 0.001), E/M ratio (*p* = 0.01), PEAC (*p* = 0.017), VAS score: Headache (*p* = 0.014), Olfactory dysfunction (*p* = 0.001), Overall burden (*p* = 0.001). Age, Lund-Mackay, Lund-Kennedy and comorbidity of allergic rhinitis or asthma were no significant difference between Eos CRSwNP and Non-Eos CRSwNP group.

**Table 1:**
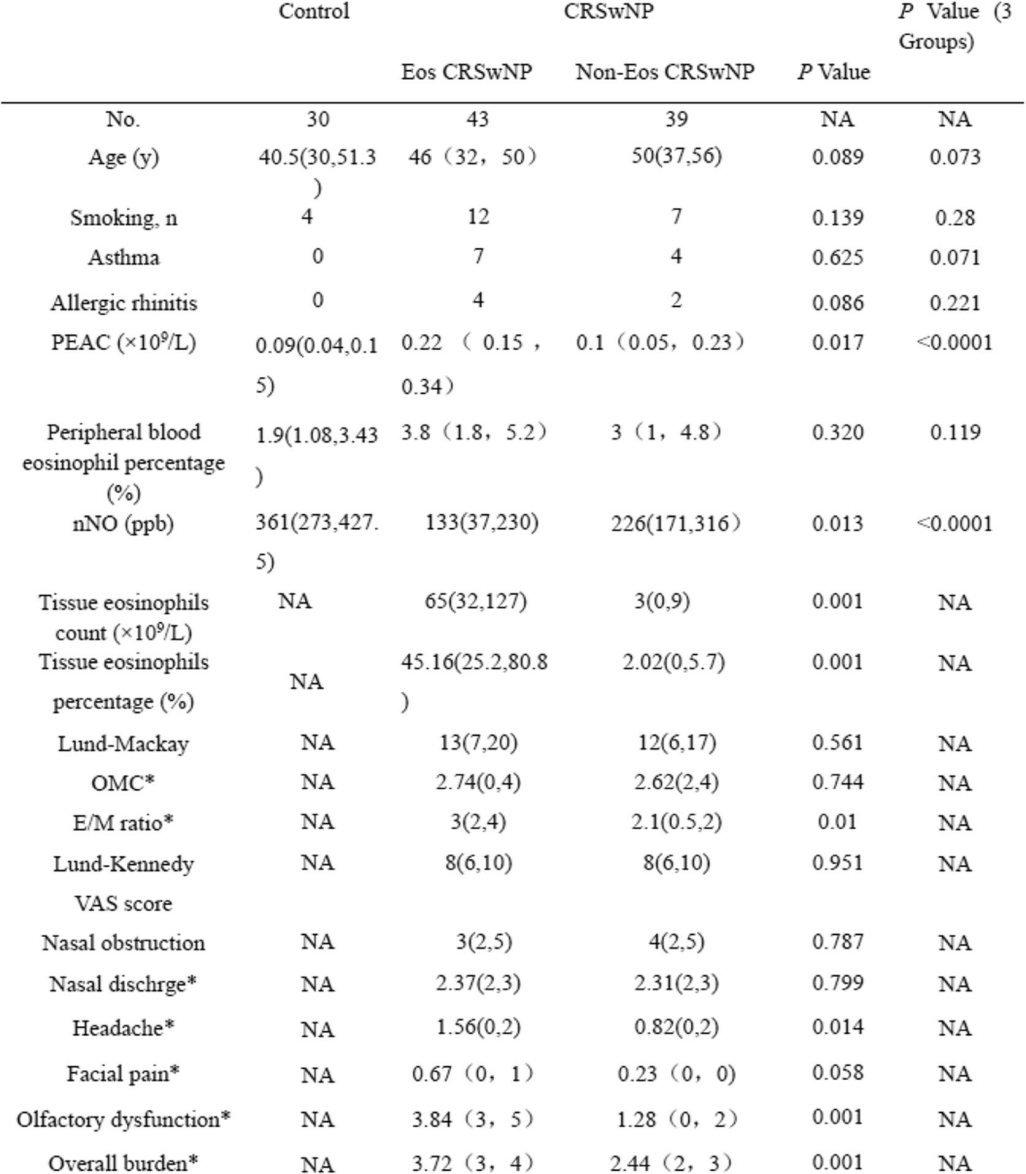
Demographic characteristics of the study population

Eos *CRSwNP* eosinophilic chronic rhinosinusitis with nasal polyps, *Non-Eos CRSwNP* non eosinophilic chronic rhinosinusitis with nasal polyps, *OMC* ostiomeatal complex, *E/M* ratio of ethmoid sinus and maxillary sinus CT score, *PEAC* peripheral blood eosinophil count, *nNO* nasal nitric oxide, *p* < 0.05 was considered statistically significant; *Data are geometric mean(25th,75th percentile)

### Nasal NO levels in patients with CRSwNP

**Fig. 2:**
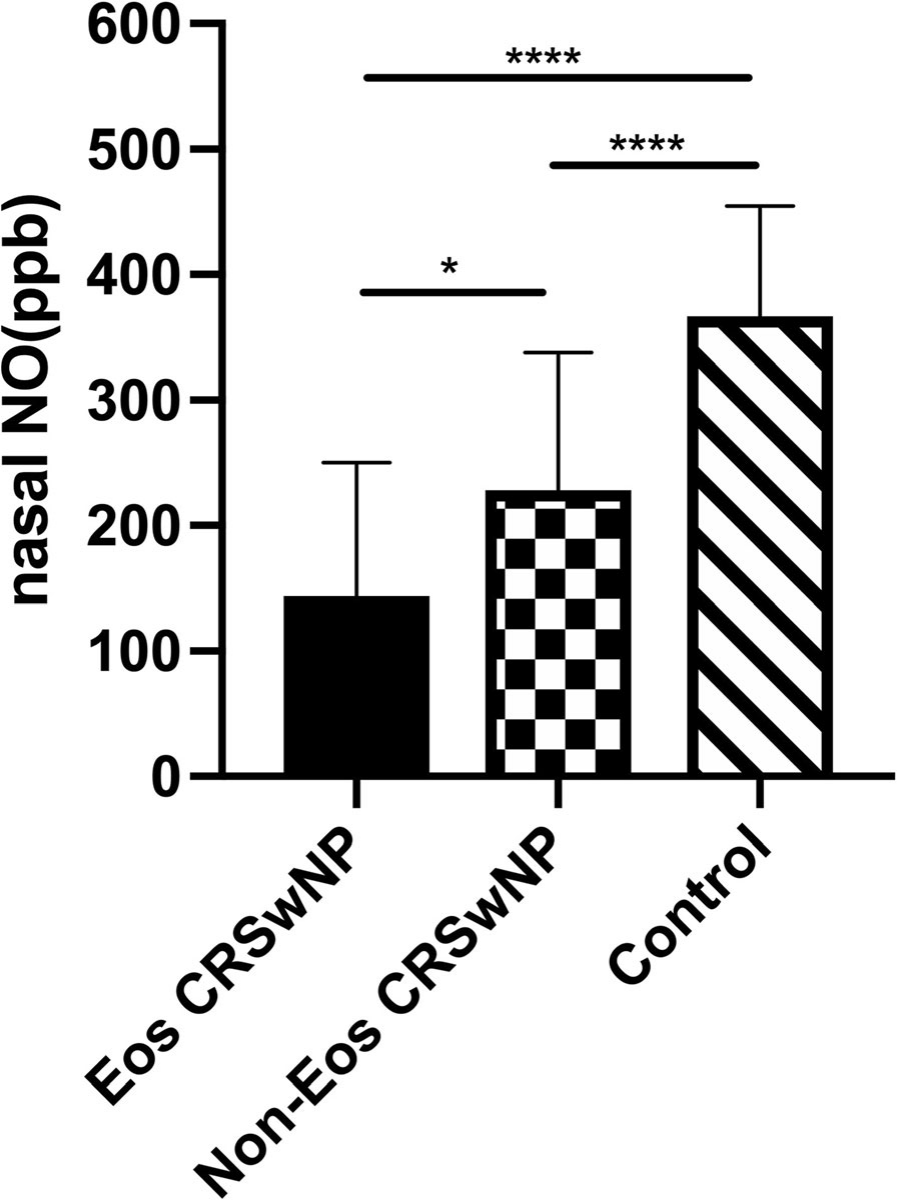
Comparison of the levels of nNO in Control, Non-Eos CRSwNP and Eos CRSwNP groups. The nNO levels of the three groups are significantly different and arranged in the following order: Control, Non-Eos CRSwNP, Eos CRSwNP. ^*^p < 0.05, ^****^p < 0.0001. Eos CRSwNP = eosinophilic CRSwNP; Non-Eos CRSwNP = non eosinophilic CRSwNP; nNO = nasal nitric oxide

Regarding nNO levels, the mean level in the Eos CRSwNP group(143.9 ± 106.2, ppb) was markedly lower than the control group (366.5 ± 88, ppb, *p* < 0.0001), while the latter was markedly higher than the Non-Eos CRSwNP group (228.3 ± 109.2, ppb, *p* < 0.0001). The mean nNO levels in the Non-Eos CRSwNP group was significantly higher than the Eos CRSwNP group (*p* = 0.013). The Eos CRSwNP group had the lowest mean nNO level (Fig. 2).

**Fig. 3:**
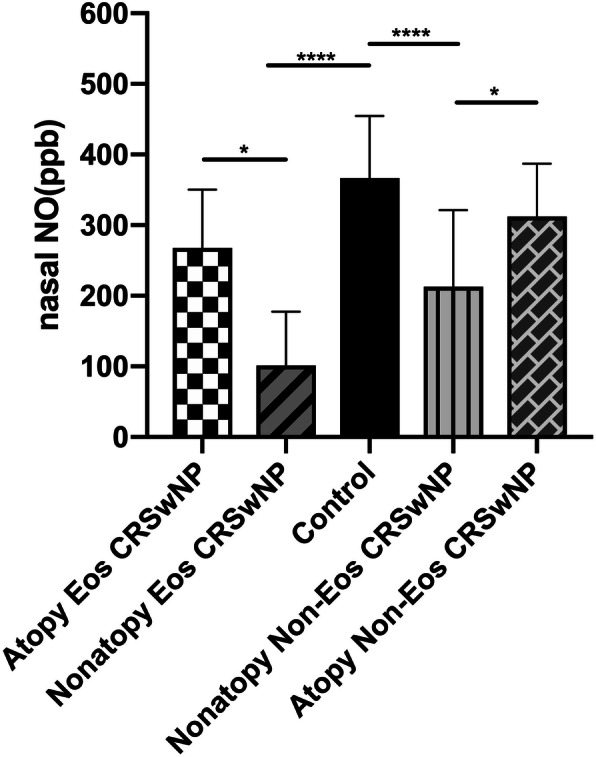
The difference in levels of nNO in patients from different groups, according to ayopic and nonatopic status. ^****^p < 0.0001, ^*^p < 0.05. nNO = nasal nitric oxide

The nNO levels of atopic Eos CRSwNP and atopic Non-Eos CRSwNP groups were significantly higher than non-atopic groups. The nNO levels of the normal control group was significantly higher than that of the atopic/ non-atopic Eos CRSwNP and non-atopic Non-Eos CRSwNP groups. However, no difference was observed in the levels of nNO compared with atopic Eos CRSwNP and atopic Non-Eos CRSwNP patients (Fig. 3).

### Factors associated with endotypes of CRSwNP

To further determine the factors associated with endotypes of CRSwNP, logistic regression analyses were conducted on the data for the two endotypes. Initially, the following variables were introduced into the univariate regression analysis model: sex, age, smoking history, peripheral blood eosinophil percentage, PEAC, Lund–Kennedy score, total VAS symptom score, Lund–Mackay score, E/M ratio, and nNO. The analysis revealed that nNO levels (OR: 1.007; 95%CI: 1.003, 1.011%; *p* = 0.001), VAS score (OR: 0.169; 95%CI: 0.085, 0.339%; *p* < 0.0001), and PEAC (OR: 0.003; 95%CI: 0.00, 0.118%; *p* = 0.002), smoking history (OR: 0.023; 95%CI: 0.075, 0.703%; *p* = 0.01) were associated with endotypes of CRSwNP (Table 2).

**Table 2:**
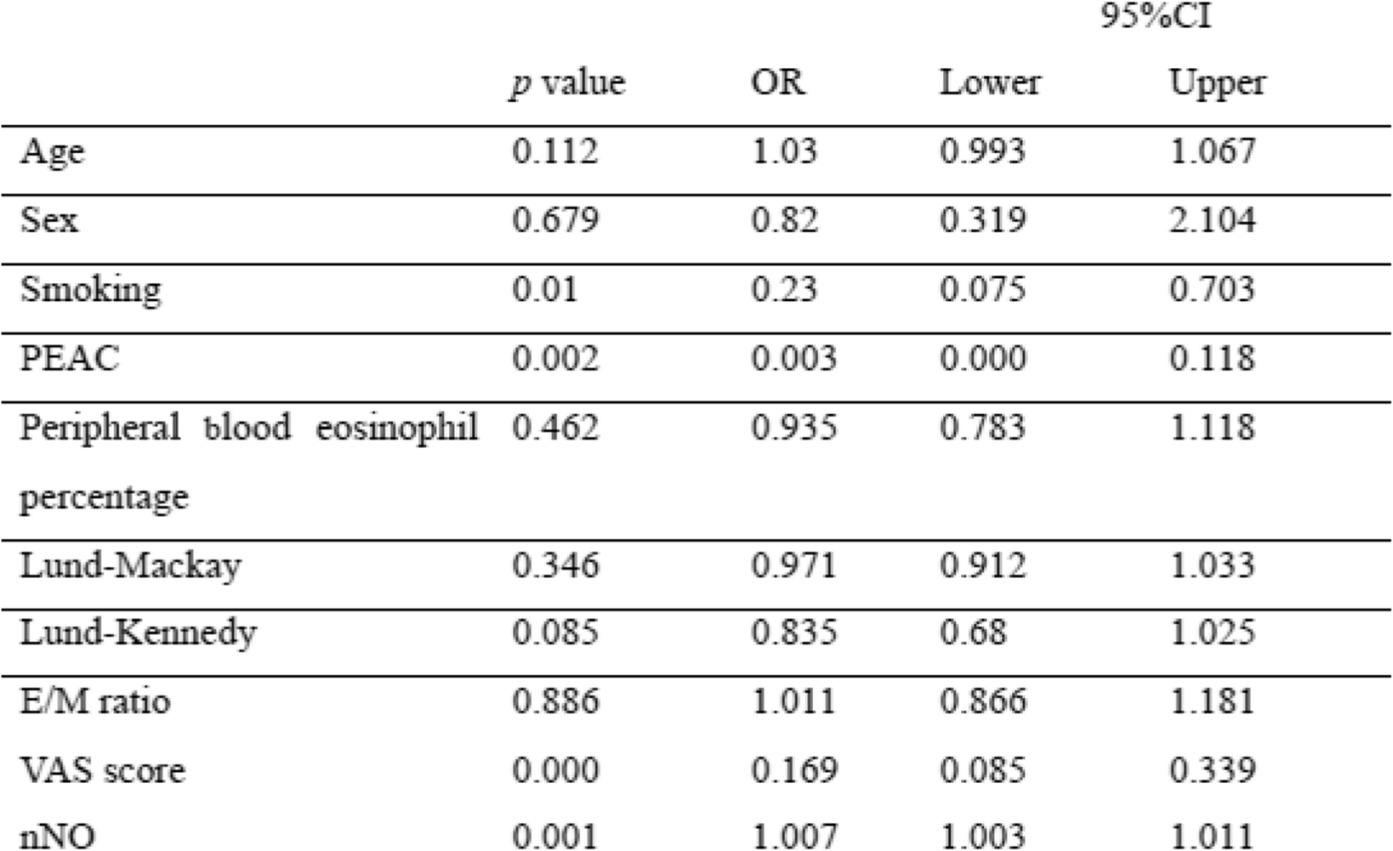
The univariate logistic regression analysis of factors associated with endotypes of CRSwNP

*E/M* ratio of ethmoid sinusand maxillary sinus CT score, *nNO* nasal nitric oxide, *OR* odds ratio, *PEAC* peripheral blood eosinophil count, *CI* confidence interval

On the basis of the univariate analyses, the variables differing between the Eos CRSwNP and Non-Eos CRSwNP groups(*p* < 0.2) were introduced into a multivariate model. The resluts confirmed that nNO level (OR: 1.01; 95%CI: 1.003, 1.016%; *p* = 0.002), PEAC (OR: 0.005; 95%CI: 0.00, 0.792%; *p* = 0.04), and total VAS score (OR: 0.158; 95%CI: 0.068, 0.367%; *p <* 0.001) were significantly correlated with endotypes of CRSwNP (Table 3).

**Table 3:**
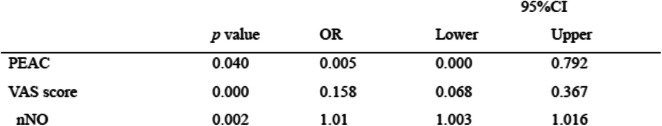
The multivariate logic regression analysis of factors associated with endotypes of CRSwNP

*PEAC* peripheral blood eosinophil count, *nNO* nasal nitric oxide, *OR* odds ratio, *CI* confidence interval

### Correlation between nNO and clinical parameters

**Fig. 4:**
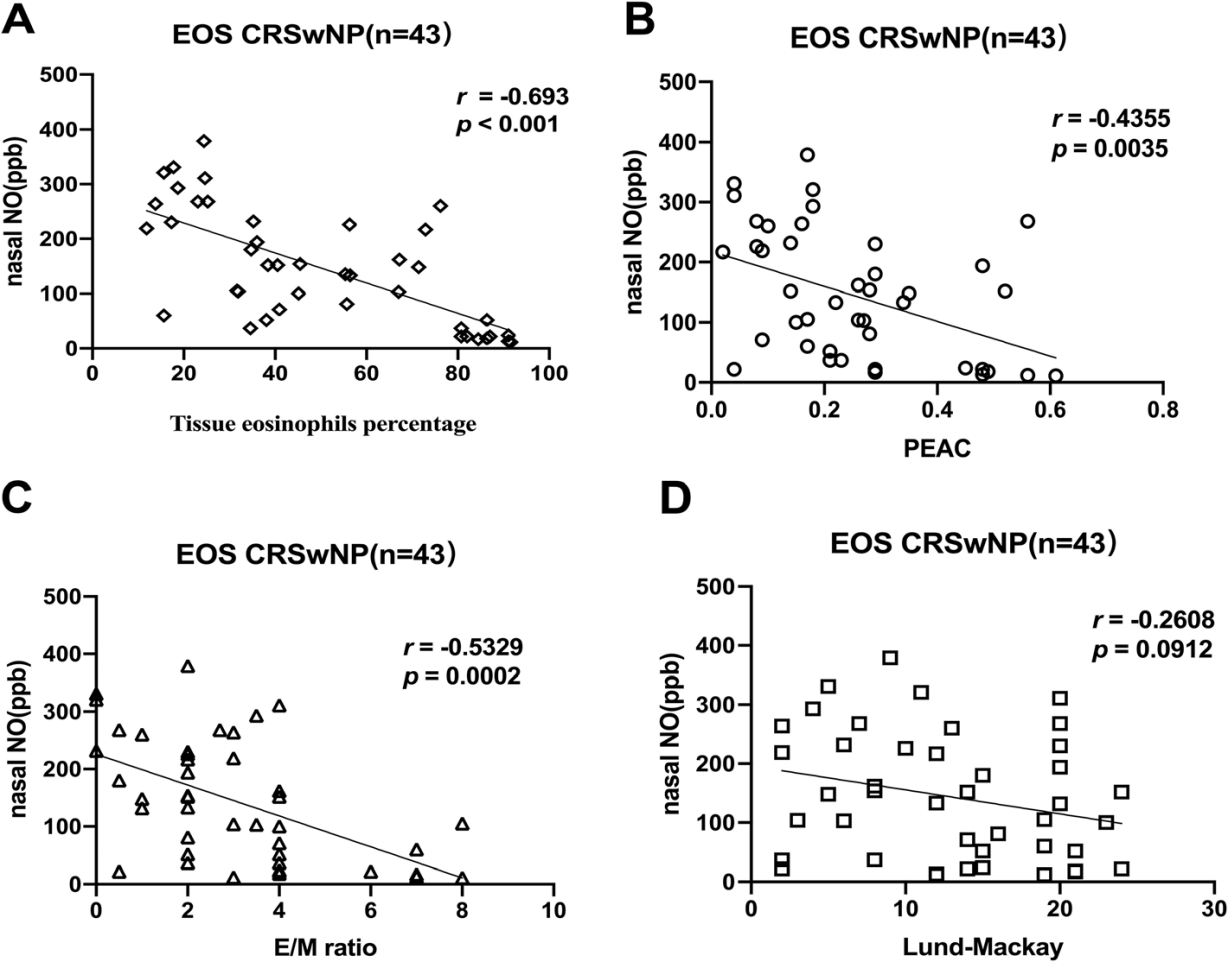
The inverse correlation was shown between nNO levels and PEAC, tissue eosinophils percentage, E/M ratio in the patients of Eos CRSwNP. Correlation between nNO levels (ppb) and (**a**) tissue eosinophils and (**b**) PEAC and (**c**) E/M ratio. Lund-Mackay score was uncorrelated to nNO levels. Correlation between nNO levels (ppb) and (**d**) Lund-Mackay score. PEAC = peripheral blood eosinophil count; E/M = ratio of ethmoid sinus and maxillary sinus CT score; nNO = nasal nitric oxide, Eos CRSwNP = eosinophilic CRSwNP

On the basis of the results of logistic regression analyses, the study explored the correlation between nNO and related clinical indicators. The tissue eosinophils percentage was negatively correlated with nNO in Eos CRSwNP patients (*r* = − 0.693, *p* < 0.001). The PEAC was negatively correlated with nNO in Eos CRSwNP patients(*r* = − 0.4355, *p* = 0.0035). Similarly, E/M ratio were also negatively correlated with nNO in Eos CRSwNP patients(*r* = − 0.5329, *p* = 0.0002). However, no significant difference was found between Lund-MacKay scores of sinus CT and nNO (*r* = − 0.2608, *p* = 0.0912). (Fig. 4).

### Predictive utility of nNO

Next, the study evaluated the predictive ability of nNO levels for diagnosis and endotypes of CRSwNP (Table 4). First, the ROC curve was used to estimate the diagnostic value of nNO. The results showed nNO had high predictive value for Eos CRSwNP (AUC: 0.939; sensitivity: 76.74%; specificity: 96.67%; cut-off value: 231 ppb; *p* < 0.001) and moderate predictive value for Non-Eos CRSwNP (AUC: 0.830; sensitivity: 63.33%; specificity: 89.74%; cut-off value: 334 ppb, *p* < 0.001). Second, the ROC curve was applied to distinguish between Eos CRSwNP and Non-Eos CRSwNP. When the nNO concentration was 158 ppb, we could discriminate Eos CRSwNP from Non-Eos CRSwNP (AUC = 0.710, sensitivity: 76.92%; specificity, 60.47%, *p* = 0.001). Moreover, when the atopic status of the patients was considered, we could distinguish atopy and nonatopy within the Eos CRSwNP and Non-Eos CRSsNP groups, respectively, based on nNO concentrations of 216 ppb and nNO concentration of 158 ppb, respectively.

**Table 4:**
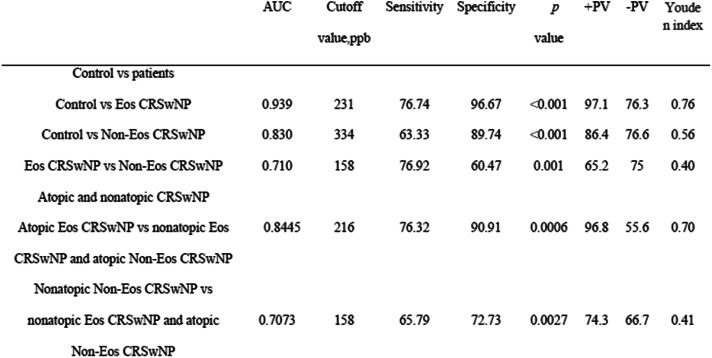
Differential diagnostic values of nNO among the control subjects and patience with Eos CRSwNP and NoN Eos CRSwNP

*ROC* Receiver operating curves, *nNO* nasal nitric oxide, *Eos CRSwNP* eosinophilic chronic rhinosinusitis with nasal polyps, *Non-Eos CRSwNP* non eosinophilic chronic rhinosinusitis with nasal polyps

**Fig. 5:**
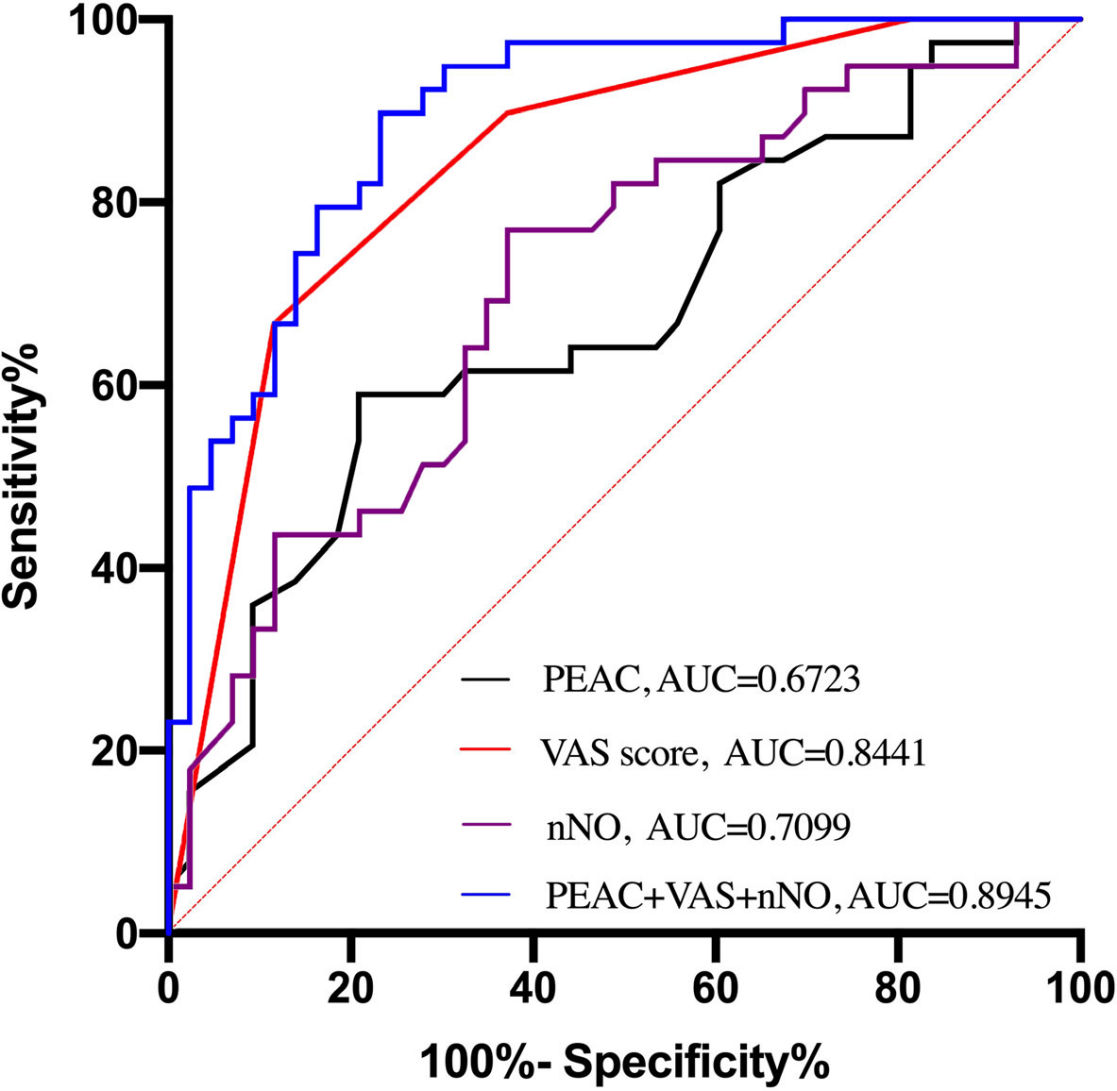
ROC curves of individual clinical predictors and joint predictors (nNO, VAS score, PEAC included, blue line) for endotypes of CRSwNP. PEAC = peripheral blood eosinophil count; nNO = nasal nitric oxide

Figure 5 shows that PEAC has low predictive value for distinguishing CRSwNP endotypes (AUC = 0.672, 95%CI = 0.56 to 0.772, *p* = 0.004, sensitivity = 79.07%; specificity = 58.97%), while VAS score has moderate predictive value (AUC = 0.844, 95%CI = 0.747 to 0.915, *p* < 0.0001 sensitivity = 88.37%; specificity = 66.67%). After it was combinated by nNO, PEAC and VAS score, the AUC was increased to 0.894 (95%CI = 0.807 to 0.951, *p* < 0.0001, sensitivity = 76.74%, specificity = 89.74%).

## Discussion

In the present study, it was observed that the nNO levels in the Eos CRSwNP and Non-Eos CRSwNP patients were significantly lower than in normal controls. Moreover, nNO levels in Eos CRSwNP patients were lower than in Non-Eos CRSwNP. These results indicated that the nNO may be a good diagnostic biomarker for Eos CRSwNP and Non-Eos CRSwNP. It was consistent with the logistic regression analysis findings. The cutoff value of nNO were used to discriminate Eos CRSwNP from Non-Eos CRSwNP is 158 ppb. In addition, PEAC and VAS scores were also related to the endotypes of CRSwNP. It was observed that the combination of nNO, PEAC and VAS scores had the highest diagnostic efficiency for distinguishing Eos CRSwNP and Non-Eos CRSwNP bying the logistic regression model and ROC curve analysis. These results clearly indicate the superiority of this joint indicator. Moreover, the AUC of 0.9391 for Eos CRSwNP was the highest among all values indicating that nNO had high predictive value for diagnosis of Eos CRSwNP. Furthermore, in the present study, it was observed that the nNO level of the atopic subgroup was significantly higher than that of the non-atopic subgroup in both the Eos CRSwNP and Non-Eos CRSwNP. It’s indicated that the atopic status of the patients influenced the levels of nNO.

In this study, PEAC was associated with the endotypes of CRSwNP, and PEAC was negatively correlated with nNO in Eos CRSwNP patients. These findings were consistent with the results of Hu et al. [19], who showed that peripheral blood eosinophil percentage and PEAC were higher in Eos CRSwNP than in Non-Eos CRSwNP patients, indicating that PEAC may play a key role in endotypes of CRSwNP. Honma et al. [20] reported that PEAC and serum IL-5 level were decreased after surgery in Eos CRSwNP patients and PEAC may reflect disease severity and prognosis after surgery. However, a few studies have suggested that various disorders and causes, including allergies, corticosteroid treatments, drug reactions, autoimmune diseases, and parasitic infections, can alter circulating eosinophil counts [21]. Therefore, the predictive ability of PEAC remains limited.

Similar to PEAC, CT examination can be employed as a marker of endotypes of CRSwNP. Ikeda et al. [5] found that CT scores were significantly higher in Eos CRSwNP compared with Non-Eos CRSwNP patients. In addition, Zhang and colleagues [22] found comparable CT scores between Chinese CRSwNP patients (neutrophil bias) and Belgian CRSwNP patients (eosinophil bias), suggesting that clinically comparable upper respiratory diseases may exist in different types of inflammation. In the present study, however, we did not found the CT score differed significantly between the two endotypes of patients. It is consistent with those of Zuo et al. [23], who concluded the nasal sinus mucosa of both Eos CRSwNP and non-Eos CRSwNP were extensively invaded, and the Lund-Mackay scores of nasal sinuses showed no significant difference. In addition, we found that E/M ratio differed significantly between the two endotypes and was associated with nNO for Eos CRSwNP patients suggesting Lund–Mackay score may play a coincidental role, rather than an E/M role in endotypes of CRSwNP. The findings of the present study were partially in accordance with the findings of Mend [9]. Similarly, Ishitoya indicated that CT scans of Eos CRSwNP patients typically show “ethmoid sinusdominant opacification,” with opacification of the olfactory cleft also being a feature [24]. Therefore, the predictive ability of CT score need more exploration.

The eosinophil infiltration in peripheral blood and nasal polyps are important indicators of local or systemic eosinophilic inflammation, respectively. Based on the negatively correlations between nNO levels and PEAC or tissue eosinophils percentage, nNO could be used to determine the severity of chronic nasal inflammatory disorders. Similarly, the level of nNO in Eos CRSwNP patients was negatively correlated with the E/M ratio, indicating that nNO could be used to determine the severity of clinical symptoms of chronic nasal inflammatory disorders.

In the present study, the Eos CRSwNP group had significantly lower nNO levels than the Non-Eos CRSwNP and control groups. In theory, large accumulations of eosinophils correspond to high levels of iNOS activity and expression, resulting in higher levels of nNO in patients with Eos CRSwNP. However, the results of our research showed the opposite findings. According to the published data, one possibility is that the polyps block the nasal cavity and reduce emission of NO [25]. Whereas, our study did not find a correlation between nNO levels and nasal polyp scores, suggesting that the lower level of nNO in the CRSwNP was not just the result of nasal meatus obstruction by nasal polyps. As Ragab et al. [26] found, increased levels of NO after surgery may reflect the role of the paranasal sinus ostial size. However, the insignificant difference between the medical groups and surgical groups suggests that the condition of the paranasal sinuses mucosa might be more important than the size of paranasal sinus ostia. Treatment of chronic sinusitis restores the expression of iNOS in the paranasal sinus mucosa and the ability of NO to pass the paranasal sinus normally. Therefore, another possibility is that Eos CRSwNP patients usually have a wide range of paranasal sinuses mucosal damage, which may lead to a decrease in nNO production [25]. All of the above suggest that nNO may be a useful measure in the monitoring of CRSwNP, since the patency of sinus ostium is not always visible during endoscopy and does not justify the use of recurrent CT scanning.

Some researches found FeNO levels in asthmatic patients are significantly positively related to the number of eosinophils in bronchoalveolar lavage fluid and induced sputum [27]. While others found the level of eosinophils in nasal polyps and peripheral blood of CRS patients was significantly negatively correlated with nNO levels [18]. So there may be differences between the paranasal sinus mucosa and the lower respiratory tract mucosa in response to eosinophilic inflammation, especially the mechanism of NO confirming this hypothesis. On one hand, the heterogeneous responsiveness of fibroblast populations in the airways to transforming growth factor-β_1_ (TGF-β_1_) and that such a heterogeneity may contribute, at least in part, to the different pathological outcomes of inflammation in the upper and lower airways [28]. On the other hand, there are differences in the structure of the upper and lower airways, and the sinus and sinus ostium may be more easily blocked.

Previous studies have found that patients with AR or athma have elevated nNO levels [29, 30]. In addition, histologic studies report increased levels of iNOS in the nasal mucosa of AR patients [31]. Liu [32] also demonstrated that CRS patients with atopy exhibited significantly higher levels of nNO compared with patients without atopy. Similarly, in the present study, it was observed that within the Eos CRSwNP and the Non-Eos CRSwNP groups, the nNO levels of the atopic subgroup were significantly higher than those of the nonatopic subgroup, respectively. These findings indicated that atopic status may influence the level of nNO. Whereas Suojalehto et al. Showed that nNO levels were lower in AR patients, and suggested that this might be due to sinus ostium obstruction [33]. The conflicting results from these studies could be attributed to the different techniques used for the determination of the levels of nNO and patient selection.

Some limitations of the present study must be addressed. Despite the present study classify CRSwNP as “eosinophilic” and “non-eosinophilic”, the endotypes of CRSwNP is still a more complex topic and major challenge. Also the sample size was relatively small and the study was conducted as a single center, which may introduce an element of bias into the study. Future studies thus should include larger sample sizes and be conducted in multiple centers. What’s more, the changes of nNO levels in postoperative patients did not discussed in this article. This will be the focus of our research in the future. In addition, large-scale routine measurement of nNO is required in clinical practice.

## Conclusion

nNO, a non-invasive, quick and easy tool, can be used to diagnose and distinguish between Eos CRSwNP and Non-Eos CRSwNP. In addition, nNO combined with blood eosinophil count and VAS score is readily available to clinicians and is valuable for the diagnosis of Eos CRSwNP. More importantly, nNO levels in CSRwNP patients may also be affected by the patient’s atopic state.

## Data Availability

The datasets used and/or analysed during the current study are available from the corresponding author on reasonable request.

## Abbreviations

95%CI: 95% confidence intervals
AUC: Area under the curve
CRSwNP: Chronic rhinosinusitis with nasal polyps
CT: Computed tomography
Eos CRSwNP: Eosinophilic CRSwNP
FeNO: Fractional exhaled NO
FESS: Functional endoscopic sinus surgery
iNOS: Inducible NOS
nNO: Nasal NO
NO: Nitric oxide
Non-Eos CRSwNP: Non-eosinophilic CRSwNP
NOS: Nitric oxide synthase
OR: Odds ratio
ROC: Receiver-operating characteristic
VAS: Visual analogue scale
E/M ratio: The ratio of the ethmoid sinus and maxillary sinus CT score
AR: Allergic rhinitis
PEAC: Peripheral blood eosinophil absolute count
